# High-Voltage Gain Single-Switch Quadratic Semi-SEPIC Converters for Powering High-Voltage Sensors Suitable for Renewable Energy Systems and Industrial Automation with Low Voltage Stresses

**DOI:** 10.3390/s25082424

**Published:** 2025-04-11

**Authors:** Frederick Nana Oppong, Soroush Esmaeili, Ashraf Ali Khan

**Affiliations:** Department of Electrical and Computer Engineering, Faculty of Engineering and Applied Science, Memorial University of Newfoundland, St. John’s, NL A1B 3X7, Canada; fnoppong@mun.ca (F.N.O.); sesmaeili@mun.ca (S.E.)

**Keywords:** boost converter, efficiency, high gain, non-isolated converter

## Abstract

This paper presents two new non-isolated DC-DC converters with and without a coupled inductor based on quadratic voltage conversion. Firstly, the coupled inductor-less type is explained in detail. It employs a voltage-boosting cell and a modified SEPIC structure to provide a high voltage boost ability with a lower and practical value for the switching duty cycle. This allows for lower power loss compared to conventional DC-DC converters. Having only one switch in the proposed converter simplifies the control and reduces the required number of control signals. Furthermore, the presented transformer-less structure can help avoid producing huge voltage spikes across the power switch. In traditional quadratic SEPIC converters, the voltage-boosting cell’s capacitor experiences relatively high voltage stress due to the voltage multiplication process. In contrast, the proposed converter offers significantly lower voltage stresses. Hence, it becomes possible to utilize a capacitor with a lower voltage rating, leading to cost savings and improved reliability and availability of suitable components. The first topology can be improved for ultrahigh voltage applications by replacing the middle inductor with a coupled transformer. Consequently, a higher voltage range with a lower switching duty cycle can be attained. Theoretical analysis and mathematical derivations are provided, and the comparison section claims the proposed converter’s ability to minimize voltage stress across the switch and output diode. Finally, experimental results are given to verify the effectiveness of the proposed converters at an output power of 260 W.

## 1. Introduction

**Figure 1:**
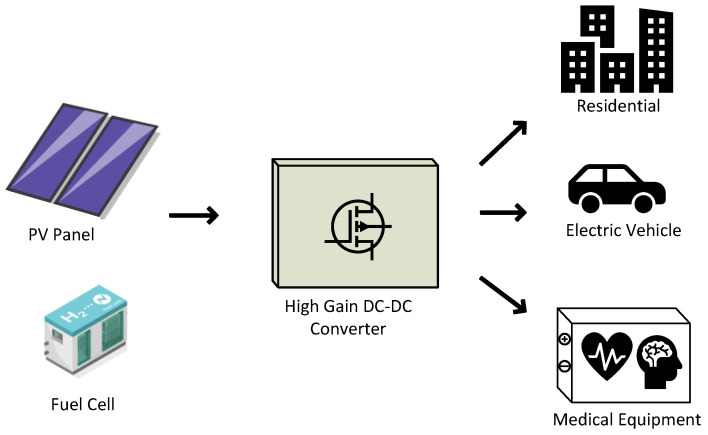
Applications of DC-DC converters.

Overexploitation of fossil fuels has led to significant environmental problems. To promote energy conservation and reduce emissions, renewable energy sources like fuel cells and photovoltaic (PV) panels are seen as viable alternatives to reduce reliance on fossil fuels. Step-up converters are commonly used in renewable energy systems to raise the voltage of the power generated by solar panels to a level that can be used by a system’s load or stored in a battery, as shown in Figure 1 [1]. LED lights and medical equipment, such as ultrasound and X-ray machines, achieve precise and stable power supplies using boost converters. Research in power electronics focuses on enhancing DC-DC boost converters with increased voltage gain, efficiency, reliability, and optimal component utilization.

Multistage DC-AC-DC conversion uses more power switches, increasing power losses and degrading efficiency. Non-isolated DC-DC converters address the drawbacks associated with isolated DC-DC converters [2,3,4,5]. These topologies offer several advantages, such as improved efficiency and performance, lower cost, compact size, and ease of control. These circuits either have common ground or separate grounds for the input and output. Common-ground non-isolated DC-DC converters enhance system performance by eliminating leakage current. The traditional non-isolated boost converter is a popular boost converter. With a voltage gain of 2.5–5, it is an attractive option for low-conversion ratio applications due to its simple configuration and reduced number of components. A high step-up converter is required to raise the low voltage output of fuel cells for use in electric vehicles and distributed power systems. Likewise, the switch and diode are subjected to high voltage stresses equivalent to the DC bus or output voltage. Despite these limitations, traditional boost converters remain popular for many applications. Besides the traditional boost converter, the SEPIC-based configurations are mainly utilized to amplify the input voltage of renewable energy sources [6]. Additionally, the SEPIC converter in [6] exhibits lower voltage stress on the switches, a non-inverting output voltage, and a continuous input current, making it suitable for renewable energy and fuel cell applications. However, the adoption of two switches increases the conduction losses. Some SEPIC converters have lower voltage gain in comparison to other known topologies like quadratic boost converters (QBCs) [7]. Furthermore, in [8], an improved SEPIC converter that can achieve a higher voltage gain is suggested. Nonetheless, the semiconductor elements experience higher voltage stresses. For further increase in voltage gain, a coupled inductor-based SEPIC converter is introduced in [9]; this single-switch SEPIC-based topology with a coupled inductor and voltage multiplier has a continuous input current. However, the coupled inductor causes the switch to experience voltage spikes.

QBCs are non-isolated DC-DC converters renowned for providing higher gain than SEPIC converters. The high voltage stress on the switch and the output diode, equivalent to the output voltage, is one of the drawbacks. Minimizing the impacts of these drawbacks has led to the development of many other QBCs [10,11,12,13,14,15,16]. Paper [10] proposes a QBC with a non-pulsating input current and a relatively low inductor current. However, it adopts two power switches, increasing the switching and conduction losses, and thereby lowering efficiency. In [11], an interleaved QBC developed from two conventional QBCs with high voltage gain is proposed. This topology also employs two switches, which increases the conduction losses and the number of passive elements. In [12], a QBC with reduced output voltage ripple and a high power density is suggested. However, the numerous switches make the control cumbersome and reduce efficiency. In [17], a QBC based on stackable switching stages is suggested to obtain a higher gain. However, the switch and the diodes experience a voltage stress equivalent to the output voltage.

Magnetic coupling is also a major technique adopted to achieve higher voltage gain in non-isolated converters [18,19,20,21,22,23]. Nonetheless, there is a voltage overshot on the switches during the turn-off process, requiring higher voltage rating switches and additional clamping circuits to be employed.

The application of the quadratic converter by incorporating switched capacitors is presented in [24]. However, the use of switched capacitors adds extra circuit elements [25]. Also, a cascaded structure can be utilized to achieve a higher step-up voltage gain [26,27,28,29,30]. Nevertheless, these structures require more switches, leading to a complex control scheme, higher switching and conduction losses, and reduced efficiency. In [31], a family of non-isolated transformer-less single-switch dual inductor high-voltage gain boost converters are introduced. However, their voltage gains are lower than those of other topologies like the QBC. With the same voltage gain, [32] uses two semiconductor switches. The additional switch reduces efficiency. In [33,34,35,36,37,38], buck–boost topologies with quadratic features have been proposed. One main challenge of designing buck–boost converters is the complexity of the control circuitry. The converter’s switching frequency and duty cycle must be carefully optimized to ensure efficient operation to avoid issues such as voltage spikes and electromagnetic interference.

Adopting the proposed high-gain DC-DC converter for sensor applications offers significant advantages, particularly in scenarios requiring efficient and stable high-voltage operation. Its transformer-less design minimizes voltage spikes and reduces power losses, ensuring reliable power delivery to sensors. The converter’s ability to achieve a high voltage boost with a practical switching duty cycle enhances its compatibility with low-voltage input sources, making it ideal for powering high-voltage sensors in renewable energy systems, electric vehicles, and industrial automation. Furthermore, the reduced voltage stress on components improves the system’s reliability and cost-effectiveness, while the single-switch configuration simplifies control, making it a practical and efficient choice for advanced sensor integration.

**Figure 2:**
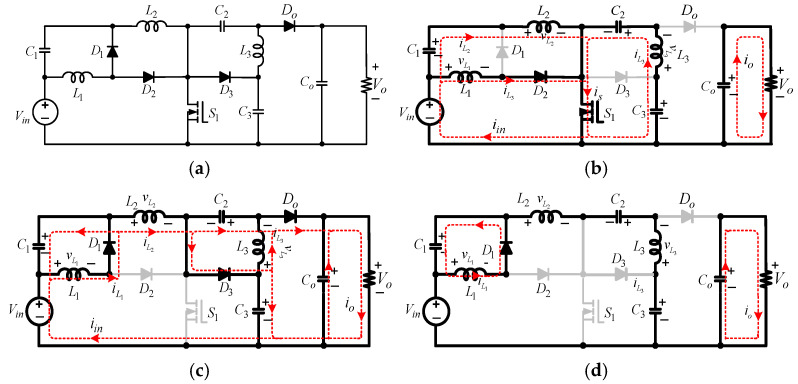
Equivalent circuits of the proposed converter without the coupled inductor. (**a**) Proposed quadratic boost DCDC converter. (**b**) Mode 1 in CCM. (**c**) Mode 2 in CCM. (**d**) Mode III in ${DCM}_{c}$.

This paper presents a novel non-isolated common-ground single-switch quadratic semi-SEPIC boost converter and its coupled inductor-based counterpart. The proposed converters are designed to operate effectively in the 100-watt to 1.2-kilowatt range, making them suitable for medium-power applications, such as battery charging systems, renewable energy interfaces, and electric vehicle auxiliaries. The proposed converter shown in Figure 2a has a high gain and a continuous input current. The proposed converter uses only one switch. Therefore, it has fewer switching and conduction losses, thereby improving efficiency. The subsequent segments of this paper are organized as follows: Section 2 offers a comprehensive description of the circuit topology of the proposed converter without a coupled inductor, encompassing fundamental principles and operational modes in CCM. In Section 3, DCM analysis is presented. In Section 4, considerations about the design of the converter components are discussed. Magnetic coupling applied to the proposed converter is studied in Section 5. Section 6 provides a comparative analysis of the proposed converter and other topologies. Experimental results are discussed in Section 7, and Section 8 concludes this work.

## 2. Proposed Quadratic Semi-SEPIC DC-DC Converter

A. Circuit Topology

The proposed non-isolated quadratic boost DC-DC converter is depicted in Figure 2a. It features one MOSFET switch ($S_{1}$), three external diodes ($D_{1}-D_{3}$), one output diode ($D_{o}$), three inductors ($L_{1}-L_{3}$), three capacitors ($C_{1}-C_{3}$), and an output capacitor ($C_{o}$). $L_{1}$ is the input inductor, and $C_{1}$ is a buffer capacitor. While $V_{in\;}$is the input source DC voltage, $V_{o}$ is the output voltage across the load, R. $D_{3}$ and $C_{3}$ provide enhanced voltage-boosting capability. The incorporation of this arrangement offers several appealing benefits, including continuous input current. The topology boasts an uncomplicated control circuitry, the ability to boost voltage to high levels, a broad range of duty cycle control, low power losses during switching, and optimal utilization of the input DC source. The switch and diodes experience less voltage stress. The common ground between the input and output eliminates leakage current.

B. Modes of Operation in CCM

The proposed converter operates in two modes. Mode 1 is when the switch is turned on and Mode 2 is when the switch is turned off for a single switching period. Analysis of the converter in continuous conduction mode (CCM) is first presented. The operational principle of the proposed converter is explained assuming the following: (a) all the components of the circuit, including inductors, capacitors, diodes, and the MOSFET switch, are ideal and have negligible series resistance; (b) any voltage drops across diodes, parasitic capacitance, and on resistance of the switch are disregarded; and (c) all capacitors are sufficiently large to maintain a consistent voltage without any variations.

*Mode 1*$\left({DT}_{s}\right)$*: See* Figure 2b. $S_{1}$ is turned on for the duty ratio, D, of the switching period, $T_{s}$. $D_{1}$, $D_{3}$, and $D_{o}$ are reverse-biased, while only $D_{2}$ is forward-biased. A closed loop is formed by $V_{in}$, $L_{1}$, $D_{2}$, and $S_{1}$. The resulting current, $i_{L_{1}}$, flows through $L_{1}$. In parallel, another closed loop is created by $V_{in}$, $C_{1}$, $L_{2}$, and $S_{1}$. The subsequent inductor current, $i_{L_{2}}$, flows through this loop. A third closed loop is created by $C_{2}$, $L_{3}$, $C_{3}$, and $S_{1}$. The inductor current, $i_{L_{3}}$, flows through this closed loop. $C_{o}$ discharges to the output. Figure 2a displays critical waveforms of the proposed quadratic converter in CCM, showing the continuous nature of all inductor currents.

By applying KVL on the first loop ($V_{in}-L_{1}-D_{2}-S_{1}-V_{in}$), $V_{L_{1}}$, across $L_{1}$ during the active time of the switch $\left({DT}_{s}\right)$ and the ensuing increasing linear current,$\;i_{L_{1}}$, is expressed as

(1) $$V_{L_{1}}=V_{in},\;\;\;\;\;\;\;\;{di}_{L_{1}}=\frac{V_{in}}{L_{1}}dt$$

For the second ($V_{in}-C_{1}-L_{2}{-S}_{1}-V_{in}$), the result of $V_{in}$ and $V_{C_{1}}$ is seen across$\;L_{2}$. Therefore, $V_{L_{2}}$ across $L_{2}$ and the corresponding linear current, $i_{L_{2}}$, are

(2) $$V_{L_{2}}=V_{in}+V_{C_{1}},\;\;\;\;\;\;\;\;{di}_{L_{2}}=\frac{V_{in}+V_{C_{1}}}{L_{2}}dt\;$$

From Figure 2b, ($C_{2}-L_{3}-C_{3}-S_{1}-C_{2}$), the voltage, $V_{L_{3}}$, developed across $L_{3}$ and its corresponding current, $i_{L_{3}}$, are expressed as

(3) $$V_{L_{3}}=V_{C_{3}}-V_{C_{2}},\;\;\;\;\;\;\;\;{di}_{L_{3}}=\frac{V_{C_{3}}-V_{C_{2}}\;}{L_{3}}dt$$

All three closed loops share the active switch, $S_{1}$. The switch current, $i_{s}$, and the input current are

(4) $$i_{s}=i_{L_{1}}+i_{L_{2}}+i_{L_{3}},\;\;\;\;\;\;\;\;i_{in}=i_{L_{1}}+i_{L_{2}}\;$$

In this mode, the capacitors’ currents, according to Figure 2b, are given by the expressions

(5) $$i_{C_{1}}=C_{1}\frac{dV_{C_{1}}}{dt}=-i_{L_{2}}=i_{L_{1}}-i_{in},\;\;i_{C_{2}}=C_{2}\frac{dV_{C_{2}}}{dt}=i_{L_{3}},\;\;\;\;\;\;\;\;\;\;i_{C_{3}}=C_{3}\frac{dV_{C_{3}}}{dt}=-i_{L_{3}},\;\;\;\;\;\;\;\;i_{C_{O}}=-\frac{V_{O}}{R}$$

*Mode 2*$(1-D)T_{s}$: See Figure 2c. $S_{1}$ is turned off and $D_{1}$, $D_{3}$, and $D_{o}$ are forward-biased, while $D_{2}$ is reverse-biased. A closed path is formed by $C_{1}$ and $L_{1}$. As a result, $V_{C_{1}}$ is built across $L_{1}$. In parallel, a closed loop is formed by $V_{in}$, $C_{1}$, $C_{2}$, $L_{2}$, and $C_{o}$. A third loop is created by $C_{2}$ and $L_{3}$. $V_{C_{2}}$ is developed across $L_{3}$. An alternative loop for $L_{3}$ is created by $C_{3}$, $L_{3}$, and $C_{o}$.

For $(1-D)T_{s}$, the voltage across $L_{1}$ and the resulting current,$\;i_{L_{1}}$, are expressed as

(6) $$V_{L_{1}}={-V}_{C_{1}},\;\;\;\;{di}_{L_{1}}=\frac{{-V}_{C_{1}}\;}{L_{1}}dt$$

The voltage developed across $L_{2}$ and its corresponding linear current, $i_{L_{2}}$, are

(7) $$V_{L_{2}}=V_{in}+V_{C_{1}}+V_{C_{2}}-V_{o},\;\;\;\;\;{di}_{L_{2}}=\frac{V_{in}+V_{C_{1}}+V_{C_{2}}-V_{o}\;\;\;\;\;}{L_{2}}dt\;$$

The electrical expressions for $V_{L_{3}}$ and the subsequent current,$\;I_{L_{3}}$, are defined as

(8) $$V_{L_{3}}=-V_{C_{2}}=V_{C_{3}}-V_{o},\;\;\;\;\;\;{di}_{L_{3}}=\frac{V_{C_{3}}-V_{o}\;}{L_{3}}dt$$

In this mode, the circuit in Figure 2c shows that the currents flowing through the capacitors can be described as follows.

(9) $$i_{C_{1}}=C_{1}\frac{dV_{C_{1}}}{dt}={i_{L_{1}}-i}_{L_{2}},\;\;\;\;\;\;i_{C_{2}}-i_{C_{3}}=C_{2}\frac{dV_{C_{2}}}{dt}={i_{L_{3}}-i}_{L_{2}},\;\;\;\;\;\;i_{C_{O}}=i_{L_{3}}-i_{C_{2}}-\frac{V_{O}}{R}$$

By applying the volt-second balance condition on $L_{1}$, $L_{2}$, and $L_{3}$, the equations below are obtained.

(10) $$\left\{\begin{array}{c} V_{L_{1avg}}=V_{in}D\;{-V}_{C_{1}}\left(1-D\right)=0\\ V_{L_{2avg}}=\left(V_{in}+V_{C_{1}}\right)D+\left(V_{in}+V_{C_{1}}+V_{C_{2}}-V_{o}\;\right)\left(1-D\right)=0\\ V_{L_{3avg}}={(V}_{C_{3}}-V_{C_{2}})D+{(V}_{C_{3}}-V_{o})(1-D)=0 \end{array}\right.$$

The voltage stresses on the capacitors and the output voltage gain in CCM are calculated. From (10), the normalized capacitor voltage stresses are as follows:

(11) $$\frac{V_{C_{1}}}{V_{in}}=\frac{D}{\left(1-D\right)},\;\;\;\;\;\;\frac{V_{C_{1}}}{V_{in}}=\frac{D}{{\left(1-D\right)}^{2}},\;\;\;\;\;\;\frac{V_{C_{3}}}{V_{in}}=\frac{1}{{\left(1-D\right)}^{2}}$$

The output voltage gain, expressed in terms of *D*, is

(12) $$\frac{V_{o}}{V_{in}}=\frac{\left(1+D\right)}{{\left(1-D\right)}^{2}}$$

Assuming $I_{in}V_{in}=I_{o}V_{o}$, applying the current second balance condition on the capacitors results in

(13) $$I_{L_{1}}=\frac{\left(1+D\right)}{\left(2-D\right){\left(1-D\right)}^{2}}I_{o},\;\;\;\;\;I_{L_{2}}=\frac{\left(1+D\right)}{\left(2-D\right)\left(1-D\right)}I_{o},\;\;\;I_{L_{3}}=\frac{1}{\left(2-D\right)}I_{o},\;\;\;I_{in}=\frac{\left(1+D\right)}{{\left(1-D\right)}^{2}}I_{o}$$

## 3. Operation of the Proposed Converter in DCM

**Figure 3:**
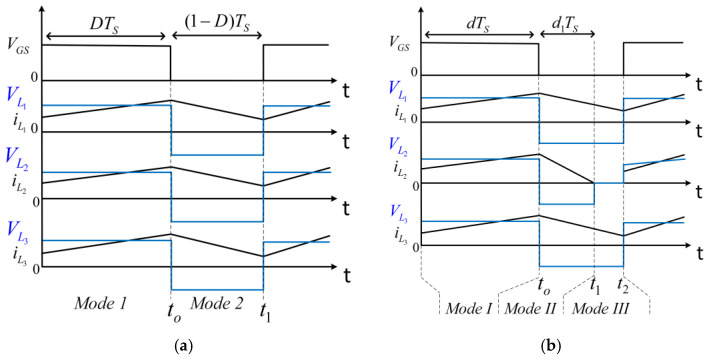

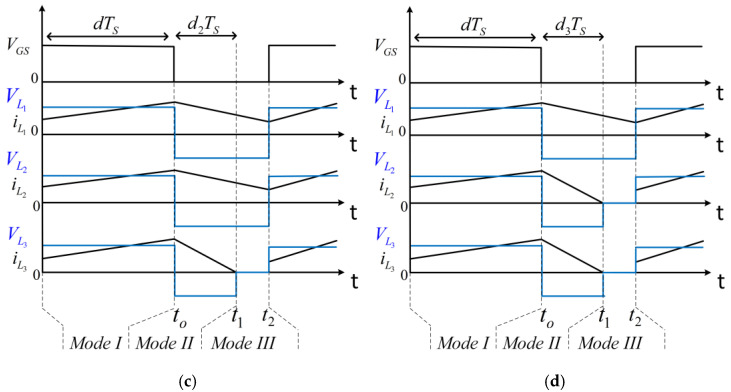
Key waveforms of the proposed quadratic converter without the coupled inductor: (**a**) CCM; (**b**) ${DCM}_{a}$; (**c**) ${DCM}_{b}$; (**d**) ${DCM}_{c}$.

In the discontinuous conduction mode (DCM), the three inductor currents, $i_{L_{1}},i_{L_{2}}$, and $i_{L_{3}}$, are considered zero at a point, making them discontinuous in the switch-off period for a switching cycle. In this regard, there are seven possible discontinuous modes. However, the current drawn by $L_{1}$ is relatively high because of the inherent voltage-boosting process, making it less probable that $i_{L_{1}}$ will enter the discontinuous region. As a result, the three more likely discontinuous scenarios are shown in Figure 3. Figure 3b shows the scenario when only $i_{L_{2}}$ is discontinuous, while $i_{L_{1}}\;$and $i_{L_{3}}$ are continuous (${DCM}_{a}$). Figure 3c shows the operation of the converter when only $i_{L_{3}}$ assumes a zero value at some point, while $i_{L_{1}}$ and $i_{L_{2}}$ are continuous (${DCM}_{b}$). In ${DCM}_{c}$ (see Figure 3d), both $i_{L_{2}}\;and\;i_{L_{3}}$ are discontinuous and only $i_{L_{1}}$ is continuous. The three discontinuous scenarios (${DCM}_{a}$, ${DCM}_{b}$, and ${DCM}_{c}$) are analyzed below.

A. Analysis of ${DCM}_{a}$

In the context of ${DCM}_{a}$, the converter operates in three distinct modes (see Figure 3b). Mode I occurs from ${0\;to\;t}_{0}$, when the switch is in the on state. This is like Mode 1 in CCM, as depicted in Figure 2a. The duty ratio for this period is d. Mode II occurs from ${t_{0}\;to\;t}_{1}$, when the switch is in the off state and $i_{L_{2}}$ is non-zero. The duty ratio for this interval is $d_{1}$. The operational circuit in Mode II is the same as Mode 2 in CCM, as shown in Figure 2c. Mode III spans from ${t_{1}\;to\;t}_{2}$, when $i_{L_{2}}$ is zero. Equations (13) and (14) detail the three inductors’ currents in ${DCM}_{a}$.


(14) $$\begin{array}{c} i_{L_{1max}}=\frac{V_{in}\;\;}{L_{1}}dT_{s},\;\;\;\;\;\;\;i_{L_{2}max}=\frac{V_{in}+V_{C_{1}}\;\;}{L_{2}}dT_{s},\;i_{L_{3}max}=\frac{V_{C_{3}}-V_{C_{2}}\;}{L_{3}}dT_{s}\;\;\; \end{array}\;\mathrm{Mode}I$$

(15) $$\begin{array}{c} i_{L_{1min}}=\frac{{-V}_{C_{1}}\;}{L_{1}}(1-d{)T}_{s},\;\;i_{L_{3}min}=\frac{V_{C_{3}}-V_{o}\;}{L_{3}}(1-d{)T}_{s},\;\;\;i_{L_{2}min}=\frac{V_{in}+V_{C_{1}}+V_{C_{2}}-V_{o}\;\;\;\;\;}{L_{2}}d_{1}T_{s} \end{array}\;\mathrm{Mode}\mathrm{II}$$

Since the inductor current, $i_{L_{2}min}$, is zero at the end of Mode II, Mode III is exempted from the mathematical analysis. The application of the volt-second balance condition on the inductors leads to the following set of equations.

(16) $$\frac{V_{C_{1}}}{V_{in}}=\frac{d}{\left(1-d\right)},\;\;\frac{V_{C_{2}}}{V_{in}}=\frac{d\left(d+d_{1}\right)}{d_{1}\left(1-d\right)},\;\;\frac{V_{C_{3}}}{V_{in}}=\frac{\left(d+d_{1}\right)}{d_{1}\left(1-d\right)},\;\;\frac{V_{o}}{V_{in}}=\frac{\left(1+d)(d+d_{1}\right)}{d_{1}\left(1-d\right)}$$

B. Analysis of ${DCM}_{b}$

In$\;{DCM}_{b}$, the three operational modes are as follows: Mode I, with duty ratio d, occurs from ${0\;to\;t}_{0};$ Mode II occurs from ${t_{0}\;to\;t}_{1}$ m, with the duty ratio $d_{2}$; and Mode III occurs from ${t_{1}\;to\;t}_{2}$, as shown in Figure 3c. Circuit illustrations of Modes I and II are identical to Modes 1 and 2 in CCM (see Figure 2). Mode I in ${DCM}_{b}$ is the same for the other DCM analysis. Equation (14) depicts all inductor currents in this mode. In Mode II, (17) describes the inductor currents. $i_{L_{3}}$ is zero in Mode III, spanning from ${t_{1}\;to\;t}_{2}$.

(17) $$\begin{array}{c} i_{L_{1min}}=\frac{{-V}_{C_{1}}\;}{L_{1}}(1-d{)T}_{s},\;\;\;\;i_{L_{3}min}=\frac{V_{C_{3}}-V_{o}\;}{L_{3}}d_{2}T_{s},\;\;i_{L_{2}min}=\frac{V_{in}+V_{C_{1}}+V_{C_{2}}-V_{o}\;\;\;\;\;}{L_{2}}(1-d{)T}_{s}\;\mathrm{Mode}\mathrm{II} \end{array}$$

The application of the volt-second balance condition on the three inductors results in (18). $V_{C_{1}}$ remains the same as expressed in (16).

(18) $$\begin{array}{c} \frac{V_{C_{2}}}{V_{in}}=\frac{d}{\left(d+d_{2}\right){\left(1-d\right)}^{2}},\;\;\frac{V_{C_{3}}}{V_{in}}=\frac{1}{{\left(1-d\right)}^{2}},\;\;\frac{V_{o}}{V_{in}}=\frac{\left(2d+d_{2}\right)}{\left(d+d_{2}\right){\left(1-d\right)}^{2}} \end{array}$$

C. Analysis of ${DCM}_{c}$

In ${DCM}_{c}$, the three operating modes are represented as follows: Mode I transpires from ${0\;to\;t}_{0}$, marked as d (duty ratio) in Figure 3d. Subsequently, Mode II occurs from ${t_{0}\;to\;t}_{1}$, also denoted as $d_{3}$ in Figure 3d. Mode III ensues from ${t_{1}\;to\;t}_{2}$. The circuit configurations corresponding to Modes I and II remain consistent with Modes 1 and 2 observed in the context of CCM (see Figure 3). Equation (14) still depicts the inductors’ currents in Mode I. At the same time, Mode II is defined by (19). Notably, inductor currents $i_{L_{2}}$ and $i_{L_{3}}$ become zero in Mode III. The circuit layout for Mode III is represented in Figure 2d.

(19) $$\begin{array}{c} i_{L_{1min}}=\frac{{-V}_{C_{1}}\;}{L_{1}}(1-d{)T}_{s},\;\;\;\;\;\;i_{L_{3}min}=\frac{V_{C_{3}}-V_{o}\;}{L_{3}}d_{3}T_{s}\;,\;\;i_{L_{2}min}=\frac{V_{in}+V_{C_{1}}+V_{C_{2}}-V_{o}\;\;\;\;\;}{L_{2}}{d_{3}T}_{s}\;\mathrm{Mode}\mathrm{II} \end{array}$$

Applying the volt-second balance condition on the three inductors results in (20). The voltage across $C_{1}$, denoted as $V_{C_{1}}$, remains unchanged and is expressed in (15). The other capacitors and the output voltages are expressed as

(20) $$\begin{array}{c} \frac{V_{C_{2}}}{V_{in}}=\frac{d}{d_{3}\left(1-d\right)},\;\;\frac{V_{C_{3}}}{V_{in}}=\frac{\left(d+d_{3}\right)}{d_{3}\left(1-d\right)},\;\;\frac{V_{o}}{V_{in}}=\frac{\left(2d+d_{3}\right)}{d_{3}\left(1-d\right)} \end{array}$$

**Figure 4:**
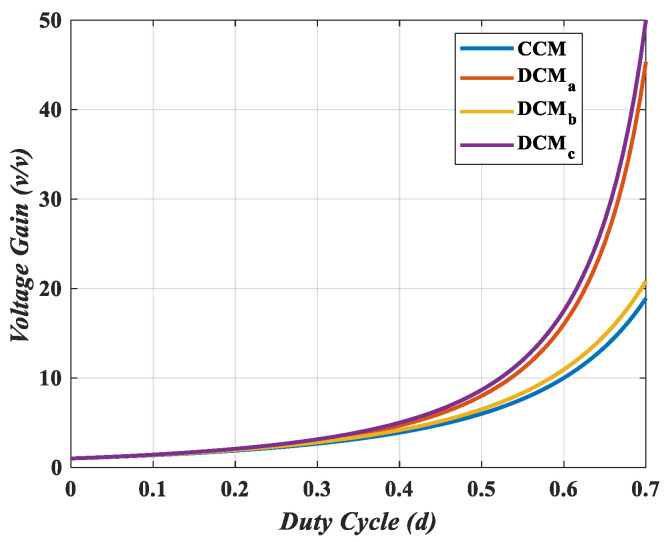
Voltage gain comparison of the proposed converter without the coupled inductor in CCM and DCM.

A comparison of the voltage gain in CCM and the three DCMs is illustrated in Figure 4. ${DCM}_{c}$ provides the highest voltage gain; however, high current flows through the inductors in DCM.

## 4. Design Specifications of the Proposed Converter

A. Inductor Design

The proposed quadratic topology has three inductors, $L_{1}$, $L_{2}$, and $L_{3}.$ Equation (1) represents the current ripple of $L_{1}$ for ${DT}_{s}.$ Therefore, the maximum value of the ripple current occurs when D=1. $L_{1}$ can be determined from (1) as

(21) $$L_{1}\geq \frac{{V_{in}}^{2}D\;}{x\%f_{s}P_{o}}\;$$

where $P_{o}$ is the output power, $f_{s}$ is the switching frequency, and x% represents the percentage factor of the ripple with respect to the load current and ranges from 10% to 20%.

From (2) and (11), the ripple current recorded through $L_{2}$ is

(22) $${∆I}_{L_{2}}=\frac{\;V_{in}\;\;}{L_{2}(1-D)}{DT}_{s}\;$$

$L_{2}$ experiences maximum ripple as D→1.

Computation for the inductor size for $L_{2}$ follows the equation

(23) $$L_{2}\geq \frac{{V_{in}}^{2}D\;}{x\%f_{s}P_{o}(1-D)}$$

The ripple current through $L_{3}$, according to (3) and (11), is

(24) $${∆I}_{L_{3}}=\frac{V_{in}\;\;}{L_{3}\left(1-D\right)}{DT}_{s}$$

Maximum ripple is experienced by $L_{3}$ as D approaches 1. The equation below serves as the basis for computing the required value of the inductor.

(25) $$L_{3}\geq \frac{{V_{in}}^{2}D\;}{x\%f_{s}P_{o}(1-D)}$$

B. Capacitor Design and Selection

The capacitors ensure the stable operation of the converter by managing voltage ripple and accommodating the required current levels. Their design involves careful consideration of parameters such as the switching frequency, input voltage, duty cycle, and the maximum allowable voltage ripple. The capacitors can be designed by considering their maximum current values and the acceptable voltage ripple. The parameter, ${∆V}_{C}$, represents the maximum allowable voltage ripple for each capacitor, usually between 1 and 10%. The voltage rating of the selected capacitors must exceed the peak voltage levels in the circuit to ensure reliability and safety. Capacitors with low ESR are preferred to minimize power loss and heat generation.

From (5) and (11), $C_{1}-C_{3}$ are designed with the expressions beneath.

(26) $$\begin{array}{c} C_{1}=\frac{I_{L_{2}}D}{f_{s}{∆V}_{C_{1}}\;}=\frac{I_{L_{2}}\left(1-D\right)}{y\%f_{s}V_{in}\;},\;\;\;\;\;\;C_{2}=\frac{I_{L_{3}}D}{f_{s}\;{∆V}_{C_{2}}}=\frac{I_{L_{3}}{\left(1-D\right)}^{2}}{y\%f_{s}V_{in}\;},\;\;\;C_{3}=\frac{I_{L_{3}}D}{f_{s}\;{∆V}_{C_{2}}}=\frac{I_{L_{3}}D{\left(1-D\right)}^{2}}{y\%f_{s}V_{in}\;} \end{array}$$

C. Semiconductor selection

The voltage, $V_{DS}$, is seen across the terminals of the switch in Mode 2 (see Figure 2c). Applying KVL results in

(27) $$V_{DS}=\;\frac{V_{o}}{\left(1+D\right)}$$

From Figure 2b, the current passing through $S_{1}$ is the sum of all inductor currents, as expressed in (4).

The current and voltage stress analysis of $D_{1}-D_{3}$ is as follows:

In Figure 2b, $D_{1}$ is off. $V_{D_{1}}$ represents the voltage across the diode terminals. Thus, from (2) and (11)

(28) $$V_{D_{1}}=\frac{\left(1-D\right)}{\left(1+D\right)}\;V_{o}$$

In Mode 2, $D_{1}$ experiences the flow of current, $i_{L_{1}}$. From Figure 2c,

(29) $$i_{D_{1}}=i_{L_{1}}$$

From Figure 2c, $D_{2}$ is reverse-biased and $V_{D_{2}}$ is the voltage developed across it. Accordingly, a deduction can be drawn from (7) and (11) as

(30) $$V_{D_{2}}=\frac{D}{\left(1+D\right)}V_{o}$$

In Mode 1 (see Figure 2b), $D_{2}$ is in the forward mode. The diode current, $i_{D_{2}}$, is expressed as

(31) $$i_{D_{2}}=i_{L_{1}}$$

Similarly, $D_{3}$ is reverse-biased in Mode 1, as displayed in Figure 2b. The reverse blocking voltage,$\;V_{D_{2}}$, and the output diode, $D_{O}$, voltage stress is computed in Mode 1 as

(32) $$V_{D_{3}}=V_{D_{o}}=\frac{V_{o}}{\left(1+D\right)}$$

## 5. Proposed Converter with Magnetic Coupling

**Figure 5:**
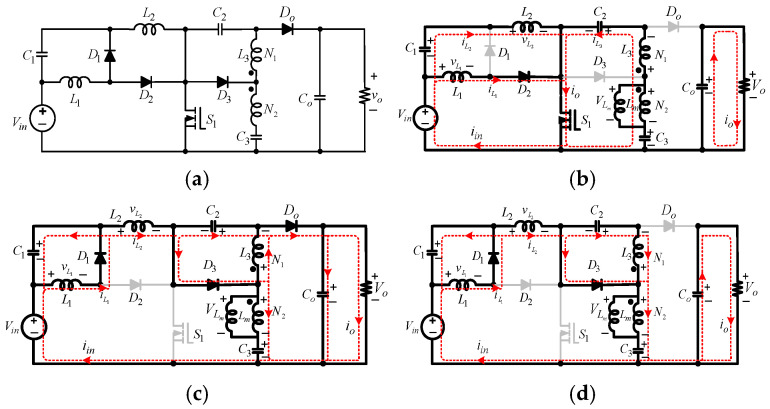
Equivalent circuits of the proposed converter with the coupled inductor: (**a**) proposed converter; (**b**) Mode A; (**c**) Mode B; (**d**) Mode C.

**Figure 6:**
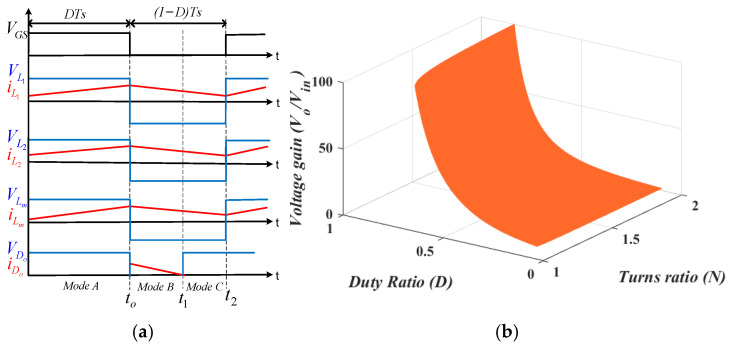
(**a**) Key waveforms of the proposed converter with the coupled inductor. (**b**) Plot of voltage gain against duty and turns ratios for the proposed converter with the coupled inductor.

To achieve a higher voltage boost and operate the converter with a lower switching duty cycle, a trans-inverse coupled inductor is employed, as can be seen in Figure 5a. The key waveforms for CCM operation are displayed in Figure 6a. The operating modes and analysis are provided below.

*A.*  Operating modes

The proposed converter with a coupled inductor operates through three distinctive modes: Mode A involves switch activation; in Mode B, the switch is turned off; and in Mode C, both the switch and the output diode are deactivated.

*Mode A*: The circuit operation involves four closed loops with different components when $S_{1}$ is active for a fraction, D, of the switching period, $T_{s}$. Only diode $D_{2}$ is forward-biased, while $D_{1}$, $D_{3}$, and $D_{o}$ are reverse-biased. Loop 1 consists of $V_{in}$, $L_{1}$, $D_{2}$, and $S_{1}.$ The input voltage, $V_{in}$, charges $L_{1}$, causing the flow of $i_{L_{1}}$ through $L_{1}$. Loop 2 consists of $V_{in}$, $C_{1}$, $L_{2}$, and $S_{1}.$ The combination of $V_{in}$ and $C_{1}$ charges $L_{2}$. This induces $i_{L_{2}}$ in this loop. Loop 3 involves $C_{2}$, $C_{3}$, $L_{3}$, and $S_{1}.$ $L_{3}$, the coupled inductor with windings $N_{1}$ and $N_{2}(with\;N=\frac{N_{2}}{N_{1}})$ is charged by $C_{2}$ and $C_{3}$. Loop 4 consists of $C_{o}$ and $R_{L}$. In this mode, the currents flowing through $L_{1}$, $L_{2}$, and $L_{m}$, all of which increase. Refer to Figure 5b for the circuit configuration.

Equation (33) represents the voltages and currents through the inductors in this mode.

(33) $$\left.\begin{array}{c} V_{L_{1}}=V_{in}\\ V_{L_{2}}=V_{in}+V_{C_{1}}\\ V_{L_{m}}=\frac{V_{C_{3}}-V_{C_{2}}\;}{N-1} \end{array}\right|\;\;\;\;\;\;\;\left.\begin{array}{c} {di}_{L_{1}}=\frac{V_{in}\;}{L_{1}}dt\;\;\\ {di}_{L_{2}}=\frac{V_{in}+V_{C_{1}}\;\;}{L_{2}}dt \end{array}\right|$$

*Mode B*: $S_{1}$ is deactivated, causing $D_{1}$, $D_{3}$, and $D_{o}$ to conduct while $D_{2}$ is off. This results in the formation of four distinct closed loops. Loop 1 involves $L_{1}$, $D_{1\;}$, and $C_{1}.$ $L_{1}$ discharges its stored energy into $C_{1}$ via $D_{1}$. Loop 2 consists of $V_{in}$, $C_{1}$, $C_{2}$, $D_{o},\;L_{2}$, and$\;C_{o}$. The input voltage and $L_{2}$ provide the energy to the capacitors in this loop. Loop 3 consists of $C_{2}$, $D_{3}$, and $N_{1}.$ $C_{2}$ is charged by the energy discharged by $N_{1}$. Figure 5c shows the circuit.

(34) $$\left.\begin{array}{c} V_{L_{1}}={-V}_{C_{1}}\\ V_{L_{2}}=V_{in}+V_{C_{1}}+V_{C_{2}}-V_{o}\;\\ V_{L_{m}}=\frac{V_{C_{3}}-V_{o}\;}{N-1} \end{array}\right|\;\left.\begin{array}{c} {dI}_{L_{1}}=\frac{{-V}_{C_{1}}\;}{L_{1}}dt\;\;\\ {dI}_{L_{2}}=\frac{V_{in}+V_{C_{1}}+V_{C_{2}}-V_{o}\;\;\;\;\;}{L_{2}}dt\; \end{array}\right|$$

*Mode C:* While $S_{1}$ is inactive, the leakage inductance of the coupled inductor causes $D_{o}$ to activate before the end of the switching period. During steady-state analysis, an ideal behavior of the coupled inductor is assumed, yet the influence of its leakage inductance is evident. This effect causes $D_{o}$ to deactivate before the switching period ends, even when the power switch is inactive. This state, displayed in Figure 5d, underscores the need for careful design of the coupled inductor, prioritizing high magnetizing inductance and minimal leakage inductance to mitigate this issue effectively. The voltage stresses on the capacitors are calculated as

(35) $$\begin{array}{c} \frac{V_{C_{1}}}{V_{in}}=\frac{D}{\left(1-D\right)},\;\;\frac{V_{C_{2}}}{V_{in}}=\frac{ND}{{(N-1)\left(1-D\right)}^{2}},\;\;\frac{V_{C_{3}}}{V_{in}}=\frac{N-1+D}{{(N-1)\left(1-D\right)}^{2}}\;\;\; \end{array}$$

The output voltage gain expressed in terms of the duty ratio is

(36) $$\frac{V_{o}}{V_{in}}=\frac{N-1+ND}{{(N-1)\left(1-D\right)}^{2}}$$

Figure 6b illustrates the voltage gain of the proposed magnetic coupling converter versus the transformer turns ratio and duty cycle. It can be observed that the voltage boost capability of the proposed converter with coupled inductors is increased by lowering the transformer turns ratio.

*B.*  Built-in regenerative snubber circuit

**Figure 7:**
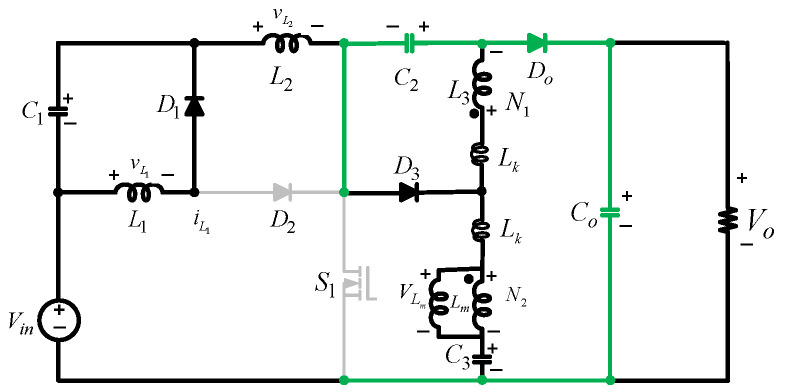
Built-in regenerative snubber circuit when the converter transitions from Mode A to Mode B.

In practice, the presence of leakage inductance can cause significant voltage spikes on the power switch due to resonance with the switch’s parasitic capacitance. These spikes can occur when the converter transitions from Mode A to Mode B when the switch is turned off. In the proposed converter, this issue is mitigated through a design that eliminates the need for an additional lossy snubber circuit. In Mode A, capacitor *C_3_* charges the coupled transformer. When the power switch turns off, Mode B starts, and the transformer is clamped to the output capacitor, releasing the stored energy from the coupled inductors and their leakage inductances to *Co*. In this mode, diodes *D_3_* and *Do* are activated, clamping the switch voltage to the difference between the output voltage and the capacitor voltage, *V_C2_*. This configuration, depicted in Figure 7, shows that the built-in regenerative snubber circuit is created using capacitors *C_2_* and *C_O_*, and the diode, *D_O_*, safely redirects the leakage energy to the power capacitors, which allows the output capacitor to absorb the energy from the leakage inductances, effectively recycling it and preventing voltage spikes on the power switch, enhancing the overall efficiency and reliability of the converter. Additionally, the presence of the DC current blocking capacitor *C_3_* in series with winding *N_2_* prevents DC current saturation of the core.

## 6. Power Loss Analysis

For power dissipation in the proposed converter, the conduction losses of the parasitic components and the switching losses of the MOSFET are the major contributors. The parasitic components, $r_{DS}$, $r_{D_{n}}$, $V_{F_{n}}$, $r_{L_{n}}$, and $r_{c_{n}}$, are the switch on-state resistance, the diode’s forward resistance, the diode threshold voltage, the inductor’s ESR, and the capacitor’s ESR, respectively.

Using (4) and (13), the conduction loss of $S_{1}$ ($P_{{con}_{s1}}$) can be expressed in the form

(37) $$P_{{con}_{s1}}={I_{{S1}_{rms}}}^{2}.r_{DS}$$

The primary source of switching losses during transitions is the capacitive turn-on loss, which arises from the discharge of the junction capacitor, $C_{oss}$, of the MOSFET. This loss is dependent on the switched voltage and the switching frequency. Typically, the capacitive turn-on energy dissipation, $E_{oss}$, can be found in the device datasheet. Therefore, the switching loss for the MOSFET can be expressed as

(38) $$P_{{sw}_{1}}=f_{s}.E_{oss}.V_{DS}=f_{s}.E_{oss}.\frac{V_{o}}{\left(1+D\right)}$$

where $I_{{S1}_{rms}}$ is the switch rms current and $f_{s}$ is the switching frequency.

The conduction loss of the diodes ${(P}_{{con}_{Dn}})$ is due to $r_{D_{n}}$ and $V_{F_{n}}$. The estimated conduction loss is

(39) $$P_{{con}_{Dn}}=I_{{Dn}_{avg}}{.V}_{F_{n}}+{I_{{Dn}_{rms}}}^{2}.r_{D_{n}}$$

The conduction losses of the inductors are estimated as

(40) $$P_{{con}_{Ln}}={I_{{Ln}_{rms}}}^{2}.r_{L_{n}}$$

The conduction losses of the capacitors take the form

(41) $$P_{{con}_{Cn}}={I_{{Cn}_{rms}}}^{2}.r_{L_{n}}\;\;$$

The inductors also experience core losses in their operation. The core loss of an inductor is calculated as

(42) $$P_{fe\left(Ln\right)}=lm\;.\;\;Ac\;\;.Kfe\;.\;{B_{max}}^{\beta }$$

where lm, Ac, Kfe, $B_{max}$, and β represent the magnetic path length, the core’s cross-sectional area, the core loss coefficient, the maximum flux density, and the core loss exponent, respectively.

## 7. Comparative Analysis

**Figure 8:**
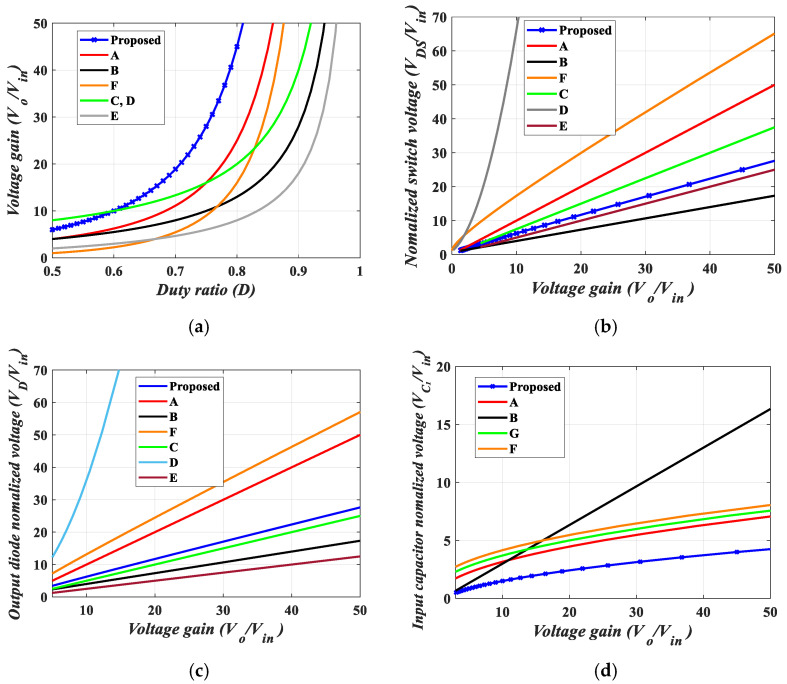
Comparison of the proposed converter with similar topologies. (**a**) Voltage gains against duty ratio. (**b**) Normalized voltage of power switches against voltage gain. (**c**) Normalized voltage of output diode against voltage gain. (**d**) Normalized input capacitor voltage against voltage gain. A: Quadratic Boost Converter (QBC), B: Ref. [2], C: Ref. [3], D: Ref. [4], E: Ref. [5], G: Ref. [9], F: Ref. [37].

The proposed quadratic boost converter was compared with other similar topologies. The analysis in terms of voltage gain, switch and diode voltage stresses, and number of elements (switches, diodes, inductors, and capacitors) is summarized in Table 1. From Table 1, it can be observed that the proposed converter has a comparable number of circuit elements. Figure 8a gives a graphical analysis of the voltage gain versus duty ratio of the proposed converter, the conventional quadratic boost converter (QBC), and other topologies. It can be observed that for D>0.6, the proposed topology provides the highest voltage gain. Furthermore, Figure 8b provides a comparison of the relationship of the normalized voltage stress on the power switches against their respective voltage gains for various topologies. In comparison to these topologies, the voltage stress on the power switch employed in the proposed converter is minimal. Similarly, the normalized voltage stress of the output diode of the proposed converter compared to that of the other stated topologies is displayed in Figure 8c. Here, again, the normalized voltage stress of the proposed converter is reasonably low. High-voltage gain DC-DC converters sometimes have high voltage stresses on capacitors. Figure 8d provides a comparison of the input capacitor voltage stress for the proposed converter and the previously mentioned topologies. The proposed converter shows the least voltage stress across the input capacitor. Thus, the proposed converter is a good candidate for applications requiring higher voltage gain and low stress on components.

**Figure 9:**
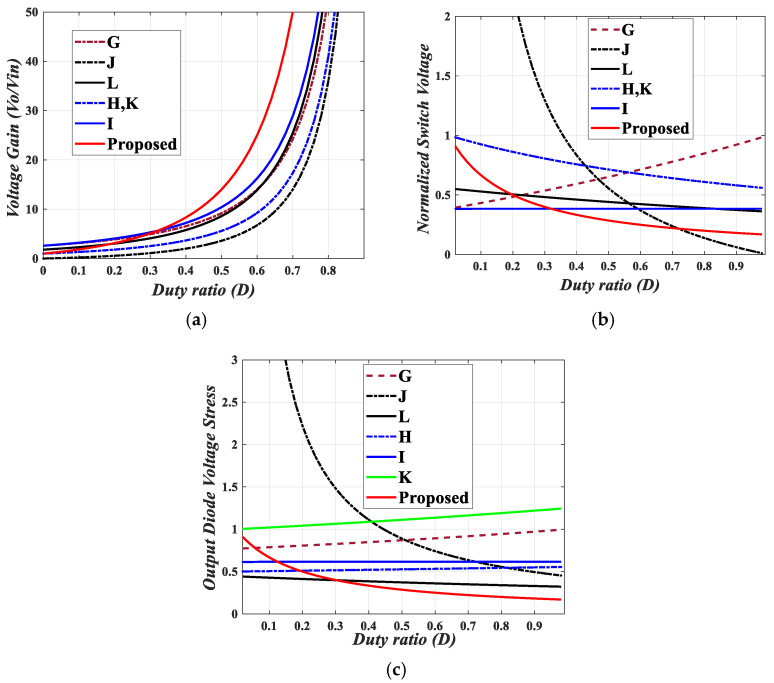
Comparison of the proposed magnetic coupling quadratic converter with similar topologies. (**a**) Voltage gains against duty ratio. (**b**) Normalized voltage of power switches against duty ratio. (**c**) Normalized voltage of output diode against duty ratio. G: Ref. [9], H: Ref. [18], I: Ref. [19], J: Ref. [20], K: Ref. [22], L: Ref. [23].

The proposed magnetic coupling quadratic converter was compared with its counterparts. For a fair comparison, we considered the same turns number for all topologies (50:40). As can be seen in Figure 9a, the proposed magnetic coupling converter provides a higher voltage gain than its counterparts for D>0.36. Figure 9a,b illustrate that the proposed converter provides the lowest voltage stress on both the power switch and the output diode compared to similar quadratic magnetic coupling topologies. This reduction in voltage stress can result in significant cost savings by allowing the use of less expensive components and decreasing maintenance needs, while also enhancing overall efficiency. Finally, Table 2 presents a brief comparison between the proposed converters and selected magnetic coupling quadratic converters.

## 8. Experimental Results

**Figure 10:**
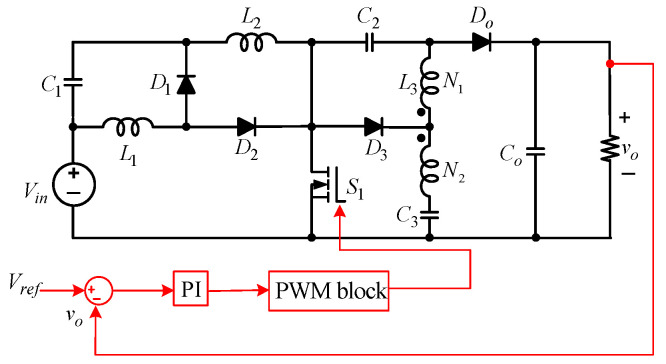
Closed-loop control of the proposed converter.

**Figure 11:**
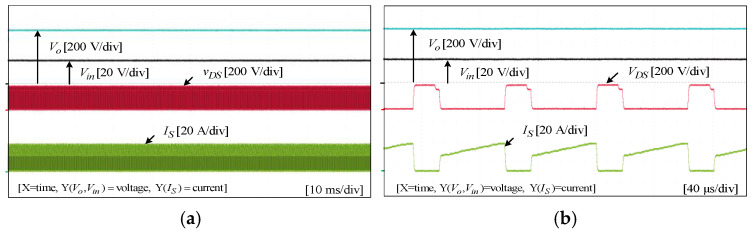
Experimental results for input and output voltages, switch drain-source voltage, and current without the coupled inductor: (**a**) 10 ms/div; (**b**) 40 µs/div.

**Figure 12:**
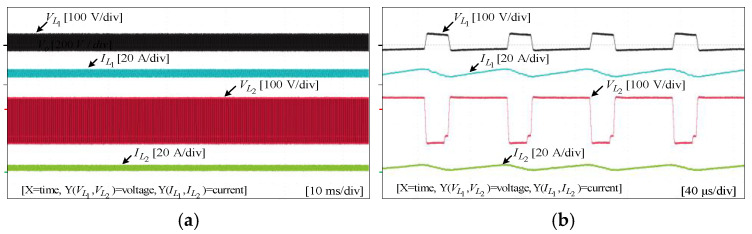
Experimental results for inductor voltages and currents of $L_{1}\;$ and $L_{2}$ without the coupled inductor: (**a**) 10 ms/div; (**b**) 40 µs/div.

**Figure 13:**
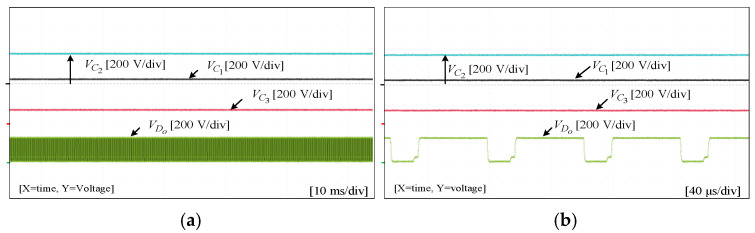
Experimental results for capacitor voltages and output diode voltage without the coupled inductor: (**a**) 10 ms/div; (**b**) 40 µs/div.

**Figure 14:**
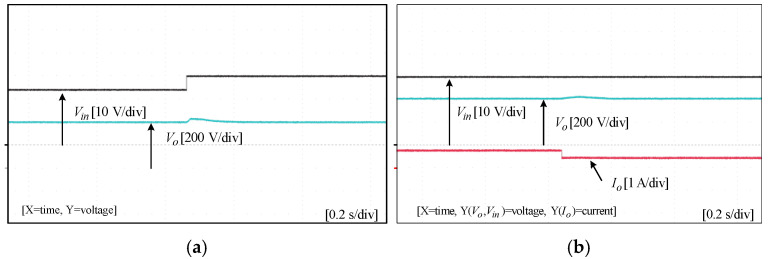
Transient experimental results without the coupled inductor: (**a**) step input voltage change; (**b**) step output load change.

**Figure 15:**
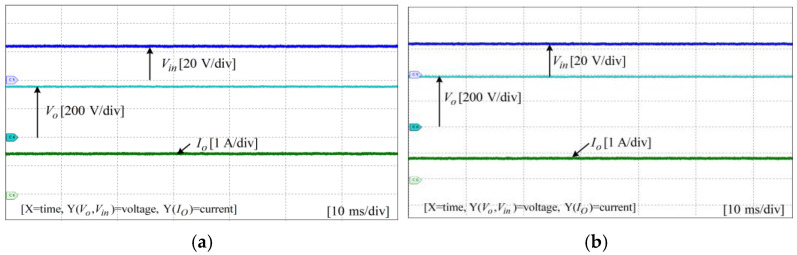
Experimental results for other loads: (**a**) RL load; (**b**) RC load.

**Figure 16:**
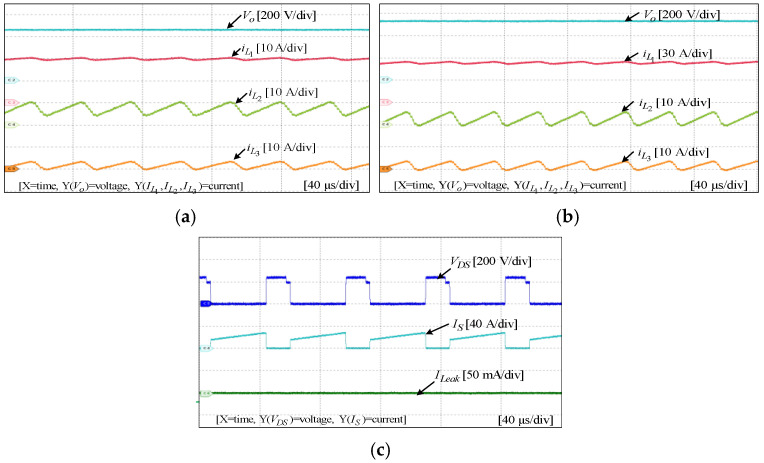
DCM experimental results for output voltage and inductor currents: (**a**) ${DCM}_{b}$; (**b**) ${DCM}_{c}$. (**c**) Recorded leakage current.

**Figure 17:**
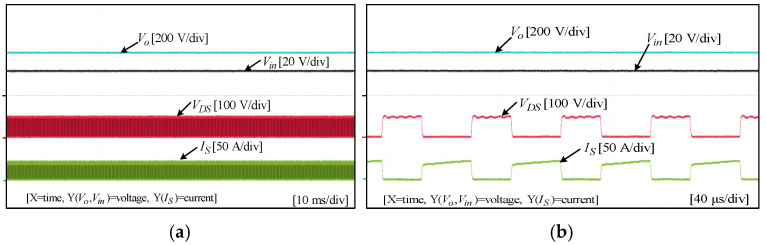
Experimental results for input and output voltages, switch drain-source voltage, and current with the coupled inductor: (**a**) 10 ms/div; (**b**) 40 µs/div.

**Figure 18:**
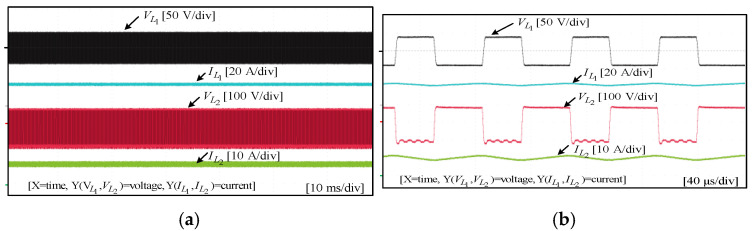
Experimental results for inductor voltages and currents of $L_{1}\;$ and $L_{2}$ with the coupled inductor: (**a**) 10 ms/div; (**b**) 40 µs/div.

To assess the performance and functionality of the proposed converter, a hardware prototype with a power rating of 260 W was developed and tested. The electrical specifications of the components utilized in the prototype can be found in Table 3. A voltage PI controller was used to analyze the closed-loop operation of the proposed converter, as depicted in Figure 10. The loop regulates the output voltage with the PI controller implemented using a digital signal processor (DSP). The outcomes of the experiments are depicted in Figure 11, Figure 12, Figure 13, Figure 14, Figure 15, Figure 16, Figure 17 and Figure 18. Figure 11, Figure 12, Figure 13, Figure 14, Figure 15 and Figure 16 show the results for the proposed converter without the coupled inductor, and Figure 17 and Figure 18 show the results obtained with the coupled inductor. In Figure 11, the input and output voltages, switch drain-source voltage ($V_{DS}$), and current ($i_{S}$) for a resistive load of 625 Ω at a switching frequency of 50 kHz and a duty ratio of 0.7 are shown. The output voltage is 400 V, with an input of 24 V. The results demonstrated that the drain-source voltage of the switch did not exhibit notable voltage stress or spikes. Figure 12 shows the voltage and current waveforms of $L_{1}$ and $L_{2}$. The inductor currents show fewer ripples and are continuous. Figure 13 illustrates the capacitor voltages and the output diode voltage stress. The input capacitor has a stress of less than 50 V, while the output diode stress is lower than the output voltage. With an increase in the input voltage from 24 V to 30 V, Figure 14a shows the closed-loop response of the converter. The duty ratio is quickly adjusted to maintain a constant output voltage of 400 V for a step input voltage change. Furthermore, Figure 14b displays the response of the converter when there is a step change in load from 625 Ω to 1250 Ω. Results for other loads tested with the proposed converter are illustrated in Figure 15. Figure 15a,b show results for an RL load (R=250 Ω, L=50 mH) and an RC load (R=250 Ω, C = 10 mF), respectively. DCM experimental results for the proposed converter are shown in Figure 16. Figure 16a,b highlight the results for ${DCM}_{b}$ and ${DCM}_{c}$. The results confirm the theoretical findings in Figure 5. To evaluate leakage current, a 1 nF capacitor was placed between the positive terminal of the input DC voltage source and the negative terminal of the output. The measurement results, presented in Figure 16c, demonstrate an insignificant leakage current, validating that the proposed inverter effectively mitigates any leakage current concerns.

**Figure 19:**
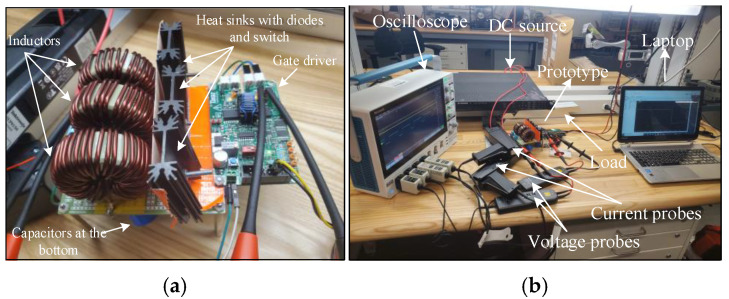
Setup pictures of the proposed converter. (**a**) Prototype only. (**b**) Whole experimental setup.

With a load of 625 Ω, the results for the input and output voltages, as well as the drain-source voltage ($V_{DS}$) and current ($i_{S}$) through the switch of the converter with the coupled inductor at a switching frequency of 50 kHz and a duty ratio of 0.54, are displayed in Figure 17. As shown, the proposed converter with the coupled inductor achieved the required output voltage at a lower duty ratio. Notably, the output voltage was maintained at 400 V, while the input voltage was set at 24 V. Figure 18 shows the voltage and current waveforms of inductors $L_{1}$ and $L_{2}$ of the coupled inductor-based converter. The prototype of the hardware and the experimental setup are displayed in Figure 19.

The experimental results not only validate the theoretical analysis but also demonstrate close alignment with the simulation findings in terms of output voltage regulation in Figure 4, switch voltage stress, inductor current behavior, and overall system response. The experimentally observed low ripple in inductor currents and minimal voltage stress on the switch corroborate the simulation, thereby confirming the accuracy of the simulated waveforms and theoretical predictions. Furthermore, the closed-loop performance shown in Figure 14a,b is consistent with simulation responses, indicating reliable controller implementation and robust converter behavior.

**Figure 20:**
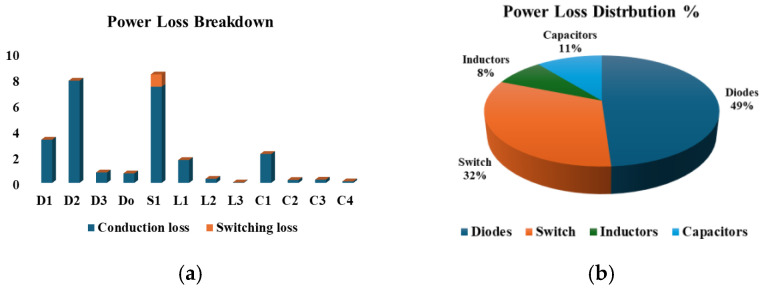
Power loss analysis of the proposed converter. (**a**) Power loss breakdown. (**b**) Components power loss distribution.

**Figure 21:**
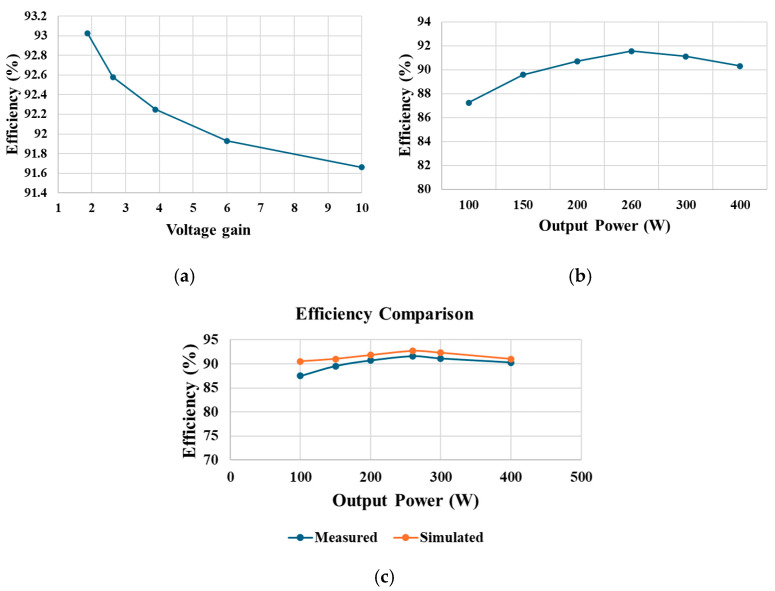
Efficiency curves. (**a**) Efficiency against voltage gain. (**b**) Efficiency against output power. (**c**) Simulated and measured efficiencies.

**Figure 22:**
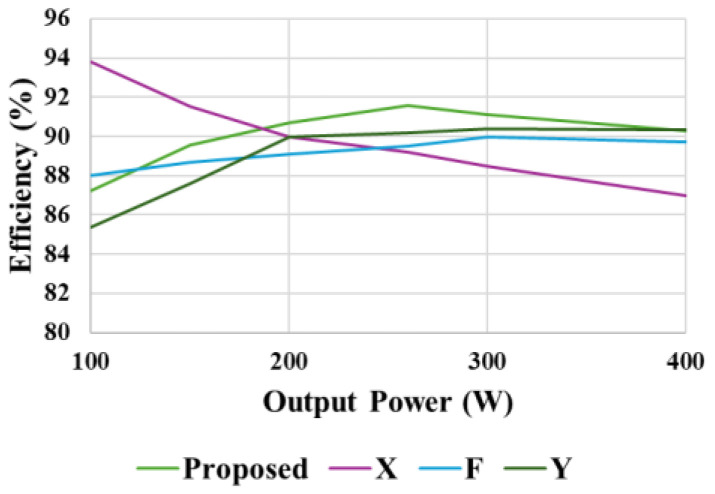
Efficiency curve comparison. X: Ref. [35], F: Ref. [37], Y: Ref. [38].

At 260 W output power, the power loss of individual components of the proposed converter carried out in PSIM is illustrated in Figure 20a. Figure 20b further provides a percentage loss distribution of the various components of the proposed converter. With a constant output power of 200 W, Figure 21a provides a relationship between voltage gain and measured efficiency. With lower gain, higher efficiency is experienced. The relationship between output power and measured efficiency is illustrated in Figure 21b. A maximum efficiency of 91.43% can be noticed at 260 W output power. Also, a comparison between the simulated and experimental efficiency of the proposed converter is illustrated in Figure 21c. While the two curves look similar, the simulated efficiency is slightly higher than the measured efficiencies. Finally, Figure 22 shows that the proposed converter has a higher efficiency compared with similar high-voltage gain converters.

## 9. Conclusions

This paper presents two innovative DC-DC quadratic semi-SEPIC boost converters with high voltage gain. These converters are adept at generating a high output voltage from a low-DC source. What truly distinguishes these converters is their unique capability to establish low voltage stress on the input capacitor and the semi-conductor elements while providing a shared ground between the source and the load. This proves highly advantageous in eradicating common-mode leakage. Furthermore, the design of this converter boasts simple control circuitry. A magnetic coupling is applied to the proposed converter to obtain the same output voltage with a lower duty ratio and low stresses on devices. These improvements collectively result in heightened overall performance. To substantiate the practicality of this proposed converter, its operational effectiveness was validated through experimental testing.

## Figures and Tables

**Table 1:** Comparison of basic features of DC-DC converters.

Description	QBC	[2]	[3]	[4]	[5]	[35]	[37]	[38]	Proposed
*G*	$\frac{1}{{\left(1-D\right)}^{2}}$	$\frac{1+2D}{\left(1-D\right)}$	$\frac{4}{\left(1-D\right)}$	$\frac{4}{\left(1-D\right)}$	$\frac{2D}{{\left(1-D\right)}^{2}}$	$\frac{3D}{\left(1-D\right)}$	$\frac{D^{2}}{{\left(1-D\right)}^{2}}$	$\frac{\left(1+2D-D^{2}\right)}{{\left(1-D\right)}^{2}}$	$\frac{\left(1+D\right)}{{\left(1-D\right)}^{2}}$
$V_{S}$	$V_{o}$	$\frac{V_{o}}{\left(1+2D\right)}$	$\frac{V_{o}}{2}$	$\frac{V_{o}}{4}$	$\frac{V_{o}\left(1-D\right)}{2D}$	$\frac{V_{o}}{3D}$	$\frac{V_{o}}{D^{2}}$	$\frac{V_{o}}{\left(1+2D-D^{2}\right)}$	$\frac{V_{o}}{\left(1+D\right)}$
$V_{Do}$	$V_{o}$	$\frac{V_{o}}{\left(1+2D\right)}$	$\frac{V_{o}}{2}$	$\frac{V_{o}}{2}$	$\frac{V_{o}}{2D}$	$\frac{V_{o}}{3D}$	$\frac{V_{o}}{D}$	$\frac{V_{o}}{\left(1+2D-D^{2}\right)}$	$\frac{V_{o}}{\left(1+D\right)}$
*N_S_*	1	1	2	2	3	1	1	1	1
*N_D_*	3	3	4	5	4	3	5	5	4
*N_L_*	2	3	2	2	3	4	3	4	3
*N_C_*	2	5	4	5	4	6	3	6	4
*N_Total_*	8	12	12	14	14	14	12	16	12

*G*: voltage gain, *V_S_*: maximum voltage stress on the power switch, *V_DO_*: maximum voltage stress on the output diode, *N_S_*: number of switches, *N_D_*: number of diodes, *N_L_*: number of inductors, *N_C_*: number of capacitors, *N_Total_*: total number of components.

**Table 2:** Comparison of magnetic coupling in quadratic converters.

Description	[9]	[18]	[19]	[22]	[23]	Proposed
*N_S_*	2	1	2	1	1	1
*N_D_*	4	5	6	6	6	4
*N_L_ + N_CL_*	1+1	2+1	1+1	2+1	1+1	2+1
*N_C_*	4	3	6	4	5	4
*N_Total_*	12	12	16	14	14	12
G	$\frac{1+D+2(\frac{1-D}{N})}{{\left(1-D\right)}^{2}}$	$\frac{1+\frac{D}{N}}{{\left(1-D\right)}^{2}}$	$\frac{1+\frac{2}{N}}{{\left(1-D\right)}^{2}}$	$\frac{1+\frac{D}{N}}{{\left(1-D\right)}^{2}}$	$\frac{\left(\frac{3D+2}{N})+(2-D\right)}{2{\left(1-D\right)}^{2}}$	$\frac{N-1+ND}{{(N-1)\left(1-D\right)}^{2}}$
$V_{S}$	$\frac{\left(1+D\right)V_{o}}{1+D+2(\frac{1-D}{N})}$	$\frac{V_{o}}{1+\frac{D}{N}}$	$\frac{V_{o}}{1+\frac{2}{N}}$	$\frac{V_{o}}{1+\frac{D}{N}}$	$\frac{(2+D(\frac{1}{N}-1))V_{o}}{\left(\frac{3D+2}{N})+(2-D\right)}$	$\frac{N(N+2)V_{o}}{N-1+ND}$
$V_{Do}$	$\frac{(1+\frac{2-D}{N})V_{o}}{{\left(1-D\right)}^{2}}$	$\frac{(1+D(\frac{1}{N}-1))V_{o}}{1+(\frac{D}{N})D}$	$\frac{\left(\frac{V_{o}}{N}\right)}{1+\frac{2}{N}}$	$\frac{(2+D(\frac{1}{N}-1))V_{o}}{1+(\frac{1}{N})D}$	$\frac{\left(\frac{{2V}_{o}}{N}\right)}{\left(\frac{3D+2}{N})+(2-D\right)}$	$\frac{N(N+2)}{N-1+ND}$

**Table 3:** Components’ electrical specifications.

Components	Values
Input voltage ($V_{in}$)	24 V
Rated output voltage ($V_{o}$)	400 V
Rated output power ($P_{o}$)	260 W
MOSFET ($S_{1}$)	NTHL065N65S3HF
Switching frequency	50 kHz
Diodes ($D_{1}-D_{4}$)	BYC30JT-600PSQ
Controller	TMS320F28335
Inductors ($L_{1}-L_{3}$)	0.176 mH
Capacitors ($C_{1}-C_{3}$)	100 μF
Output Capacitor ($C_{o}$)	100 μF

## Data Availability

Data is contained within the article.
